# The evolution of insect visual opsin genes with specific consideration of the influence of ocelli and life history traits

**DOI:** 10.1186/s12862-022-01960-8

**Published:** 2022-01-07

**Authors:** Quentin Guignard, Jeremy D. Allison, Bernard Slippers

**Affiliations:** 1 Department of Zoology and Entomology, Forestry and Agricultural Biotechnology Institute, University of Pretoria, Pretoria, 0002 South Africa; 2 Natural Resources Canada, Canadian Forest Service, Great Lakes Forestry Centre, 1219 Queen Street E, Sault Ste. Marie, ON P6A 2E5 Canada; 3 Department of Biochemistry, Genetics and Microbiology, Forestry and Agricultural Biotechnology Institute, University of Pretoria, Pretoria, 0002 South Africa

**Keywords:** Opsin evolution, Ocelli, Colour vision

## Abstract

**Background:**

Visual opsins are expressed in the compound eyes and ocelli of insects and enable light detection. Three distinct phylogenetic groups of visual opsins are found in insects, named long (LW), short (SW) and ultraviolet (UV) wavelength sensitive opsins. Recently, the LW group was found to be duplicated into the LW2b and the LW2a opsins. The expression of LW2b opsins is ocelli specific in some insects (e.g., bees, cricket, scorpion flies), but the gene was not found in other orders possessing three or less ocelli (e.g., dragonflies, beetles, moths, bugs). In flies, two LW2b homologs have been characterised, with one expressed in the ocelli and the other in the compound eyes. To date, it remains unclear which evolutionary forces have driven gains and losses of LW opsins in insects. Here we take advantage of the recent rapid increase in available sequence data (i.e., from insect genomes, targeted PCR amplification, RNAseq) to characterize the phylogenetic relationships of 1000 opsin sequences in 18 orders of Insects. The resulting phylogeny discriminates between four main groups of opsins, and onto this phylogeny we mapped relevant morphological and life history traits.

**Results:**

Our results demonstrate a conserved LW2b opsin only present in insects with three ocelli. Only two groups (Brachycera and Odonata) possess more than one LW2b opsin, likely linked to their life history. In flies, we hypothesize that the duplication of the LW2b opsin occurred after the transition from aquatic to terrestrial larvae. During this transition, higher flies (Brachycera) lost a copy of the LW2a opsin, still expressed and duplicated in the compound eyes of lower flies (Nematocera). In higher flies, the LW2b opsin has been duplicated and expressed in the compound eyes while the ocelli and the LW2b opsin were lost in lower flies. In dragonflies, specialisation of flight capabilities likely drove the diversification of the LW2b visual opsins.

**Conclusion:**

The presence of the LW2b opsin in insects possessing three ocelli suggests a role in specific flight capabilities (e.g., stationary flight). This study provides the most complete view of the evolution of visual opsin genes in insects yet, and provides new insight into the influence of ocelli and life history traits on opsin evolution in insects.

**Supplementary Information:**

The online version contains supplementary material available at 10.1186/s12862-022-01960-8.

## Background

The visual system is vital for the survival of insects [1]. Visual cues and signals mediate the location and acquisition of resources, mates and avoidance of danger [2]. Insects perceive light primarily with three main organs; compound eyes and ocelli in the adult stage and stemmata in the larval stage. The diversification of visual opsins is one route for the development of novel spectral sensitivities [1, 3]. Visual opsins are conserved proteins and group into three major clades linked to the wavelength they are most sensitive to [4, 5]. The three groups are known as long (~530 nm), short (~ 440 nm) and ultraviolet (~350 nm) wavelength (LW, SW and UV, respectively) visual opsins [2, 6].

Insects usually possess at least one copy of the LW, SW and UV opsins. Lacking a copy of one or more LW, SW or UV opsins has often been linked to a particular life history or biology. For example, the Neuropteroidea (Strepsiptera, Coleoptera, Raphidioptera, Neuroptera) and the American cockroach *Periplaneta americana* have lost their SW opsin and the corresponding blue photoreceptor, likely due to their ancestor being nocturnal [5, 7]. The number of visual opsins was found to be reduced in three subterranean beetles; *Neobidessodes gutteridgei* and *Paroster macrosturtensis* have lost their visual opsins completely, while *Limbodessus palmulaoides* has lost all but the LW opsin [8]. Conversely, duplication of visual opsins in dragonflies (Odonata) has been linked to the prominent size of their eyes and the role of vision in the ecology (i.e., prey location and capture) of this order [9].

Two distinct groups of LW opsins differentially expressed between the ocelli and the compound eyes have previously been identified. A homolog of the LW opsin was found to be ocelli specific in bees [10], scorpionflies [11], the cricket *Gryllus bimaculus* [12], the cockroach *P. americana* [13], dragonflies [9] and flies [14, 15]. Ocelli specific opsins evolved at a slower rate than the other LW opsins in bees and formed a separate clade from the rest of LW opsin sequences from the same order [10–12, 16]. Recent phylogenetic analyses focused on the Pancrustacea including a few insect species and could separate these two clades of LW opsins in insects, namely LW2a and LW2b [17]. The LW2b group contained most of the previously described ocelli specific opsins and LW2a most of the LW opsins expressed in the compound eyes. However, both groups contained ocelli specific opsins and the link between the two groups of LW opsins and the presence or absence of ocelli in insects remained unclear.

The link between the LW2b opsin and the ocelli remains poorly understood. In flies, two LW2b homologs have been characterised, with one being expressed in the ocelli and the other one in the compound eyes and the larval stemmata [14, 15]. In dragonflies, different opsins were specifically expressed in the ocelli, the compound eyes and the larvae but subsequent phylogenetic analyses did not group all the ocelli specific opsins together [9]. In Hymenoptera, one exception was found where the LW2b opsin was expressed in the compound eyes instead of the ocelli in a fig pollinator [16]. No LW2b opsins were described in some insects possessing ocelli, such as mayflies [18], beetles [7] and true bugs, moths and butterflies [19]. To date, it is uncertain if the LW2b is missing in some groups of insects due to a lack of data or a gene loss.

The increased availability of insect genomes (GenBank [20], I5K [21], VectorBase [22], Flybase [23], Hymenopteran Genome Database [24]) offers a large amount of data that remains unexplored with respect to broader evolutionary relationships of visual opsin across Insects. In this study, we characterize the occurrence and the phylogenetic relationship of all available sequences of visual opsins from public data. Specifically, we (i) identified, extracted and compiled data sets of DNA sequences of the visual opsin genes from gene sequences or genomes from 18 insect orders, (ii) determined the phylogenetic relationships of the extracted visual opsin gene sequences, (iii) link this more complete view of opsin phylogenetic groups to classifications of visual opsin genes, and (iv) consider how the loss of ocelli and key life history traits may have shaped the evolution of opsin genes.

## Results

**Fig. 1:**
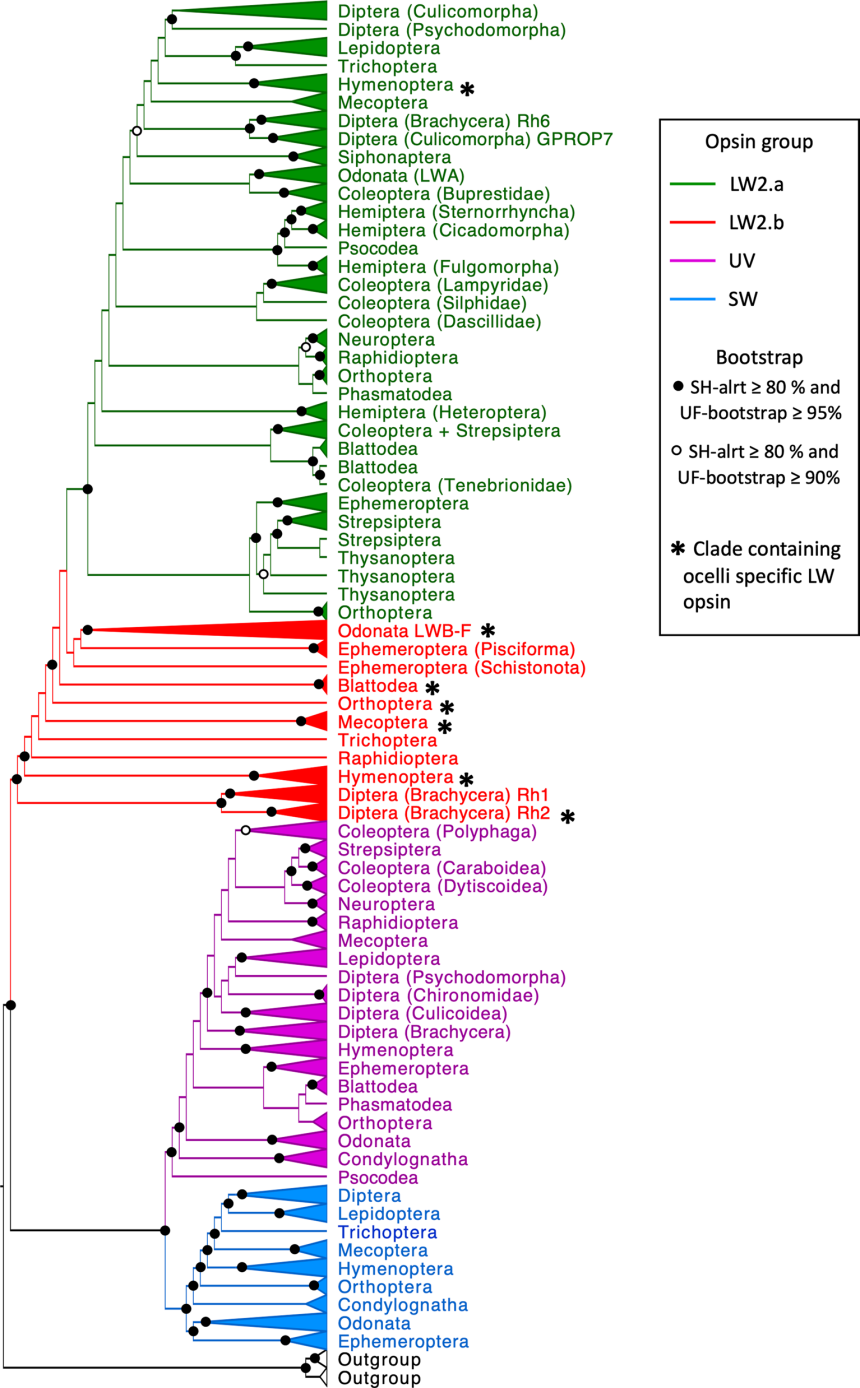
Maximum-likelihood tree of 1000 insect visual opsins sequences. The LW2b, LW2a, SW and UV opsins are red, green, blue and purple, respectively. Branches were collapsed to the highest rank when species grouped together within the same family, suborder or order. Node circles indicates UFbootstrap and SH-alrt value, only nodes over SH-alrt support ≥ 80% and UF-bootstrap ≥ 95% (solid circles) and SH-alrt support ≥ 80% and UF-bootstrap ≥ 90% (open circle) are represented

This study found a total of 1000 insect visual opsin sequences of 340 amino acids in length after alignment (Additional file 1: Table S1). These opsins came from 18 orders of insects, including 36 sub-orders, 89 families and 218 species. The data collected was from RNA or DNA extractions (i.e., PCR or sequencing). In some cases, the available set of opsins was incomplete as the objective of the study that generated the data was to target specific visual opsins. The maximum-likelihood phylogenetic tree (Fig. 1 and Additional file 2: Fig. S1) of the visual opsins contained three well separated (SH-alrt ≥ 0.8/UFBoot ≥ 0.95) major clades that corresponded to the LW, SW and UV visual opsin genes. The LW, SW and UV opsin clades contained 565, 187 and 248 opsin sequences, respectively.

A total of 34 groups of LW2a opsin gene sequences were found (Fig. 1, green nodes). All LW2a opsin genes group together within the orders Lepidoptera, Trichoptera, Hymenoptera, Mecoptera, Siphonaptera, Neuroptera, Raphidioptera, Phasmatodea, Odonata (LWA), Ephemeroptera and Psocodea. Two clades or more were found in Coleoptera, Hemiptera, Orthoptera, Blattodea, Thysanoptera, Diptera and Strepsiptera. For each of the Thysanoptera, Strepsiptera and Blattodea orders, insects from the same order are all closely related. The LW2a opsins of Diptera are divided into two clades, both well supported. The first Dipteran LW2a opsin clade contained the *Rh6*/*GPROP7* opsin present in all the Dipteran suborders. The second LW2a opsin clade of Diptera contained the *GPROP1-6*-like opsins only present in the suborder of Culicomorpha and Psychodomorpha. The LW2a opsins of Hemiptera divided into two clades; one containing the Heteroptera suborder and the second one containing the Fulgomorpha, Cicadomorpha and Sternorrhyncha suborders. The Coleoptera are separated in four groups that contains different families. The SW opsin group is divided into nine clades containing species from the same order or higher ranks. The UV opsin is divided into 20 clades where species from the same order group together, with the exception of Coleoptera, Diptera and the Condylognata (Hemiptera + Thysanoptera) that are present in more than one clade.

A copy of the LW2b opsin (Fig. 1, red nodes) was found in nine insect orders including the Diptera (Brachycera), Hymenoptera, Raphidioptera, Trichoptera, Mecoptera, Ephemeroptera, Odonata, Orthoptera and Blattodea. The LW2b opsin lineage contains the ocelli specific opsins found in five species of Hymenoptera, all Odonata (*LWD* and *LWE*), the field cricket *G. bimaculus*, four Mecoptera, the cockroach *P. americana* and the common fruit fly *Drosophila melanogaster* (Rh2). A duplication of the LW2b opsin was found in all the Diptera (named *Rh1* and only found in Brachycera) except for *D. suzukii* and *Glossina morsitans,* and in all Odonata (*LWB-C-F*).

**Fig. 2:**
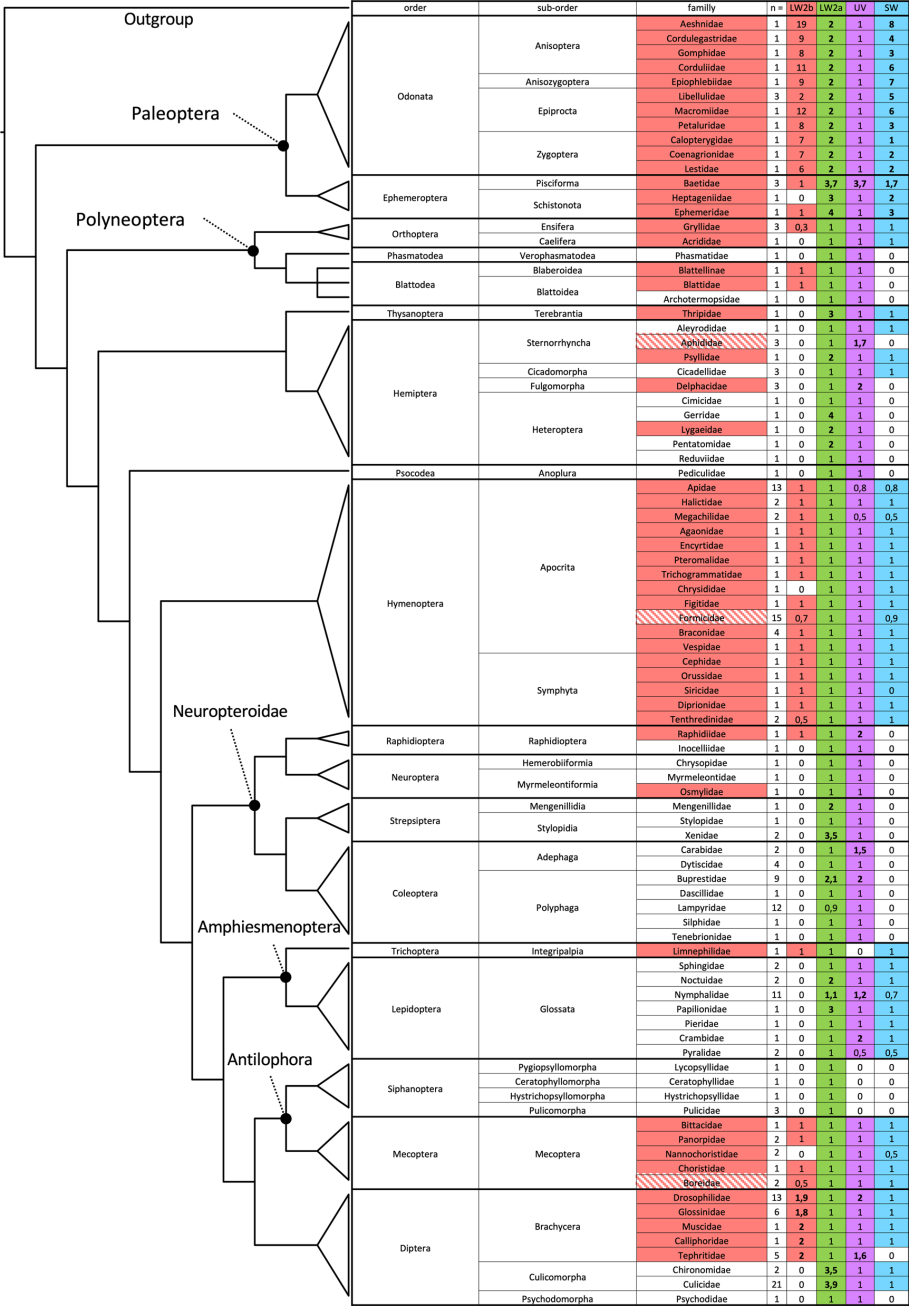
Evolutionary history of the number of visual opsins in 89 families of Insects (n = number of species for each family). Number of LW2b, LW2a, SW and UV opsins were averaged per family. Boxes are highlighted in red, green, blue and purple respectively for the LW2b, LW2a, SW and UV opsins when the average number of opsin per species within each group was higher than zero. Bold numbers indicate an average number of visual opsin per species ≥ 1. Families are highlighted or dashed in red when the presence of three ocelli in the adult stage was consistent or variable, respectively. The illustrated insect phylogeny (left) was manually coded following the insect phylogeny from Misof et al. [27]

All species that had a copy of the LW2b opsin also possessed three ocelli in at least one morph, life stage or caste (Fig. 2). Three ocelli and one copy of the LW2b opsin was found in the Ephemeroptera (except *Epeorus* sp. EP006), Odonata, Orthoptera (except *Locusta migratoria*, *Dianemobius nigrofasciatus*, and *Schistocerca gregaria*), Hymenoptera (except *Chrysis viridula*, *Camponotus atriceps*, *Cataglyphis bombycinus*, *Cerapachys biroi*, and *Tenthredo koehleri*) and the one species of Trichoptera (*Limnephilus lunatus*). In the Raphidioptera, Blattodea and Diptera a copy of the LW2b opsin was found within families possessing three ocelli. No copy of the LW2b was extracted from Mecoptera (Nannochoristidae) larvae, the one Thysanoptera (*Frankliniella occidentalis*) examined and in ant workers. The presence of three ocelli varied within Hemiptera and Neuroptera, but the LW2b opsin was never found. A strong and significant relationship between the presence of 3 ocelli and the presence of LW2b was observed (χ^2^ = 126.05, df = 1, p-value < 2.2 × 10^–16^).

All species possessed one copy of the LW2a opsin, with the exception of *Camponotus rufipes* and *Microphotus* sp*.* Multiple copies of the LW2a opsin were found in the order Thysanoptera, in the sub-order Culicomorpha (Diptera), in the families Psyllidae, Gerridae, Lygaeidae and Pentatomidae (Hemiptera), Mengenillidae and Xenidae (Strepsiptera), Buprestidae (Coleoptera), Noctuidae, Nymphalidae and Papilionidae (Lepidoptera). No significant relationship between the presence of 3 ocelli and the presence of LW2a was observed (χ^2^ = 1.9157 × 10^–31^, df = 1, p-value = 1).

The SW opsin was identified in 55 families of insects, but not found in the Phasmatodea, Blattodea, Psocodea, Raphidioptera, Neuroptera, Strepsiptera, Coleoptera and Siphanoptera, in the sub-orders of Fulgomorpha and Heteroptera (Hemiptera), Psychodomorpha (Diptera) and in the families Siricidae (Hymenoptera) and Tephritidae (Diptera). Duplication of the SW opsin was found in all the Palaeoptera (Odonata and Ephemeroptera) families except for one species of Calopterygidae (Odonata). Within the 32 families where the LW2b opsin was found, 27 also possess a copy of a SW opsin. A significant but weak relationship between the presence of 3 ocelli and the presence of SW was observed (χ^2^ = 33.822, df = 1, p-value = 6.038 × 10^–09^).

The UV opsin was found in every order except for Trichoptera and Siphanoptera. Duplication of the UV opsin occurred in the families Baetidae (Ephemeroptera), Aphididae and Delphacidae (Hemiptera), Raphidiidae (Raphidioptera), Carabidae and Buprestidae (Coleoptera), Nymphalidae and Crambidae (Lepidoptera) and Drosophilidae and Tephritidae (Diptera). No significant relationship between the presence of 3 ocelli and the presence of UV was observed (χ^2^ = 1.7211, df = 1, p-value = 0.1896).

## Discussion

This study used 1000 visual opsins from 18 insect orders to determine the phylogenetic relationships among insect opsins. It provides the most comprehensive analysis of the evolution of visual opsin genes in insects to date. The LW2b opsin was described in new insect orders (e.g. Odonata, Blattodea, Thysanoptera, Raphidioptera and Tricoptera) and is shown to only occur in insects with three ocelli. Some insects with three ocelli and all insects with less than three ocelli were found to lack the LW2b opsin. Our phylogenetic tree is the first to strongly link all but one of the previously described ocelli specific opsins in the LW2b opsin group. Dragonflies and flies were the only insects to possess more than one copy of the LW2b opsin, likely linked to their life history traits.

The presence of a LW2b opsin in insects with thee ocelli adds evidence to its hypothesised function in insect flight. All but one ocelli specific opsin previously described [9–15] belong to the LW2b opsin group in our trees, confirming that LW2b opsins are likely ocelli specific in most insects. The LW2a and LW2b appeared before the radiation of hexapods and are not present in the Crustacea [17, 25]. Two important morphological differences that distinguish Insecta from Crustacea are the fused median ocellus [26] and the ability to fly in most adults [27]. Three ocelli are primarily found in insects that are strong fliers [28] and facilitate detection of horizon tilt and a fast head and body reaction to altitude changes [29]. New flight capabilities likely drove the ocelli specific LW2b opsin to adapt to its function in flying insects. It is worth noting that in many ways swimming by crustaceans and flying by insects presents similar visual ecologies and challenges which complicates the interpretation of the relationship between flight abilities and LW2b opsins.

The LW2b opsin gene was commonly found in insects together with a SW opsin gene. The loss of SW opsin was found to be linked with a nocturnal lifestyle in the American cockroach *P. americana* [13] and the praying mantis *Tenoda sinensi* [30], adults not feeding such as *Sirex noctilio* [31] or due to a nocturnal ancestor in the Neuropteroidae [7]. Most LW2b opsins found in insect ocelli absorb in the green part of the light spectra, and are often expressed with an UV opsin and/or photoreceptor [12, 30, 32, 33]. Diurnal insects use the contrast between the green foliage and UV sky landscape as a primary source of information for landmark navigation [34]. The positive relationship between the SW and LW2b opsins suggests an important role of the LW2b opsin in higher light levels likely for daytime activities, and perhaps a role in flight stabilisation.

The LW2b opsin likely has alternative functions other than flight stabilisation as it was found in some flightless insects. A LW2b opsin gene was found in the nocturnal and reluctant flier, the American cockroach. In addition to flight stabilisation, ocelli were found to be linked with celestial navigation [35–37], light polarisation [37], circadian rhythm [16, 38] and detection of variation in light intensity that mediates flight activity [39]. Similarly, males of a fig pollinator species do not fly, spend all their life inside the fruit (in low light conditions) and do not have ocelli, yet a LW2b opsin is expressed in this species [16]. In agreement with previous studies [17], opsin expression is flexible and can switch between compound eyes and ocelli, likely to adapt to functions other than flight stabilisation. Two good examples of flexibility in opsin expression are flies and dragonflies where a duplication of the LW2b opsin was found, with one homolog being ocelli-specific and one or more LW2b homologs expressed in the larvae and/or the compound eyes.

In Diptera, two LW2b homologs were only found in higher flies (Brachycera) and no LW2b opsins were found in lower flies (Culicomorpha and Psychodomorpha). The ancestor of Diptera was believed to have three ocelli and aquatic larvae similar to the lower dipteran sub-orders [40]. The absence of LW2b opsin in lower flies is most likely due to the lack of ocelli in these sub-orders. The various LW2a homologs found in the lower flies *Aedes aegypti* (*GPROP1-6*-like gene) are first expressed in the aquatic larvae and then in the compound eyes of the adults [41, 42]. All but one LW2a homologs (*Rh6*) are absent in higher flies, but the ocelli specific LW2b (*Rh2*) was conserved with the three ocelli. It is likely that higher flies duplicated the conserved LW2b *Rh2* opsin expressed in their ocelli to create the LW2b homolog *Rh1*, expressed both in the terrestrial larvae and in the adult compound eye [15]. In agreement with previous studies [12, 43], the split between *Rh1* and *Rh2* in our phylogenetic tree is more recent than the split between the different Dipteran sub-orders, supporting the interpretation that the *Rh1* appeared after the emergence of higher flies. Therefore, the historically named blue-green opsin group of Diptera can be considered as a LW2b opsin which has undergone a duplication, likely driven by the transition from an aquatic to a terrestrial larval life style.

In dragonflies, most of the LW opsin expansion resulted in the diversification of numerous LW2b opsins. Our results correlate with previous studies showing a segregation between two LW opsin groups in dragonflies [17]. The LW2a and LW2b genes in the dragonfly *Ladona fulva* are found in two different genomic regions [9]. The authors of that study demonstrated that only one to two LW2b opsins were found to be ocelli specific while the other LW2b homologs were expressed in the compound eyes and larval eyes. The high demand for visual performance and flight capabilities in dragonflies, in addition to the aquatic larvae, likely drove the diversification of opsins in this group. In various insects, it is the LW2a opsin that is duplicated and adapted to the specific needs of each species [7, 18, 19, 44]. It is unclear why the LW2b opsins in dragonflies evolved and diversified instead of the LW2a.

## Conclusions

This study confirmed that the LW2b opsin is well conserved among insects possessing three ocelli. The LW2a genes in our study grouped differently within and between orders, but are usually poorly supported. Our study supports a paraphyly of the LW2b opsin as previously shown [4, 5, 9, 10, 12, 16, 17, 33] over a monophyly ([11] and reconciliation tree in [17]). These differences could be the results of the trimming of the sequences. That LW2b is ocelli-specific opsin is consistent with a potential role in flight stability and/or horizon detection in flying insects. We hypothesize that life history and transition to a terrestrial environment in fly larvae was a driving force in opsin evolution in Diptera and could explain the historically named blue-green clade unique to flies, in a broader evolutionary scenario. Similarly, dragonflies also evolved numerous homologs of the LW2b opsins likely as a part of the evolution of more accurate flight capabilities in addition to their life history traits. Many species across various insect orders possess three ocelli but no LW2b opsin was found (e.g. Orthoptera, Hemiptera, Thysanoptera or Neuroptera). It is possible that this is due to a lack of data in these species, and this needs to be confirmed. If not, these species offer opportunities to further investigate the different function of the LW2b and LW2a opsins in the ocelli.

## Methods

To determine the relationships among visual opsins in insects, we identified opsin DNA, RNA and protein sequences via searches of the literature [4, 5, 7, 9, 10, 12] and online databases ((GenBank [20], I5K [21], VectorBase [22], Flybase [23], Hymenopteran Genome Database [24]) (Additional file 1: Table S1). We performed BLAST searches for visual opsin DNA, RNA or protein sequences using annotated genes from *Apis mellifera* and *Drosophila melanogaster* to find insect visual opsins when genomes were available. Sequences with an e-value < 10^–40^ were retained. We used the genomic sequences to locate opsin genes in the unpublished *Sirex noctilio* genome (Alisa Postma, pers. comm). The *Rh7* opsin was excluded from the dataset due to the uncertain role of this opsin in vision [4]. The gene predictor Augustus (http://bioinf.uni-greifswald.de/augustus/submission.php) was used to translate DNA sequences into amino acid sequences. Sequences of RNA were translated into amino acid sequences via MEGA7 [45]. Five onychophoran opsins were chosen as an outgroup (OG) as they are a sister group of arthropod visual opsins [46].

Amino acid sequences were aligned using MAFFT using the default parameters. After alignment, data were visually curated to remove remaining intron sequences. Aligned amino acid sequences were cut and sequences between the K^25^ and the A^329^ of the *A. mellifera Rh1* LW (sequence annotated relative to the *Bos taurus* rhodopsin) were retained. Curated and aligned sequences are available (see section “Availability of data and materials”). The phylogenetic reconstruction was performed using IQtree v.1.4.4 [47]. The most probable amino acid substitution model was found to be the LG + F + I + G4. This model was used to build an independent maximum-likelihood tree with an ultra-fast bootstrap value of 1000 ultrafast bootstrap iterations (UFboot, Minh et al. [48]) and a 1000 Shimodaira-Hasegawa approximate likelihood-ratio test (SH-alrt, [49]) to assess the nodal support. Graphic representation of phylogenetic relationships was done using Figtree (V.1.4.3 http://tree.bio.ed.ac.uk/software/figtree).

The number of opsins found in each opsin group, as defined in the maximum-likelihood phylogenetic tree, were determined for each species and averaged for each family of insects. The presence or absence of three ocelli in the adult life stage of each species was determined from the literature (Additional file 3: File S1). In instances where there was no information available for a given species, we assumed that species within families shared the same number of ocelli as adults.

### Statistical analyses

A correlation between the presence/absence of a LW2b opsin and three ocelli in the adult stage was tested with a Chi-square test. The number of opsins from each of the four groups were transformed in “presence” or “absence” for every individual and tested against the presence of three ocelli. In some cases, available data from RNA were extracted from a younger stage (e.g. larvae in *Nannochorista philpotti*, *Nannochorista dipteroides*) or different caste (e.g. worker in ants) that do not possess three ocelli. For these individuals, the presence of three ocelli was labelled as “absent” at adult stage in this analysis.

## Supplementary Information

**Additional file 1: Table S1.** Visual opsins from online sources and literature used for phylogenetic analyses, including accession numbers and references.

**Additional file 2: Fig. S1.** Complete maximum-likelihood tree of 1000 insect visual opsin sequences. The LW2b, LW2a, SW and UV opsins are red, green, blue and purple, respectively. Node circles indicates UFbootstrap and SH-alrt value.

**Additional file 3: File S1.** Literature describing the number of ocelli in various insect orders.

## Data Availability

The aligned sequences and the phylogenetic trees analysed during the current study are available in the open Harvard dataverse repository at: https://dataverse.harvard.edu/dataset.xhtml?persistentId=doi:10.7910/DVN/FVLIBE.

## Abbreviations

LW: Long-wavelength sensitive opsin
SW: Short-wavelength sensitive opsin
UV: Ultraviolet-wavelength sensitive opsin
