# Supplementary figures and images for: The evolution of insect visual opsin genes with specific consideration of the influence of ocelli and life history traits
Source: BMC Ecol Evol. 2022 Jan 7;22:2. doi: 10.1186/s12862-022-01960-8 (PMC8739693; doi:10.1186/s12862-022-01960-8)

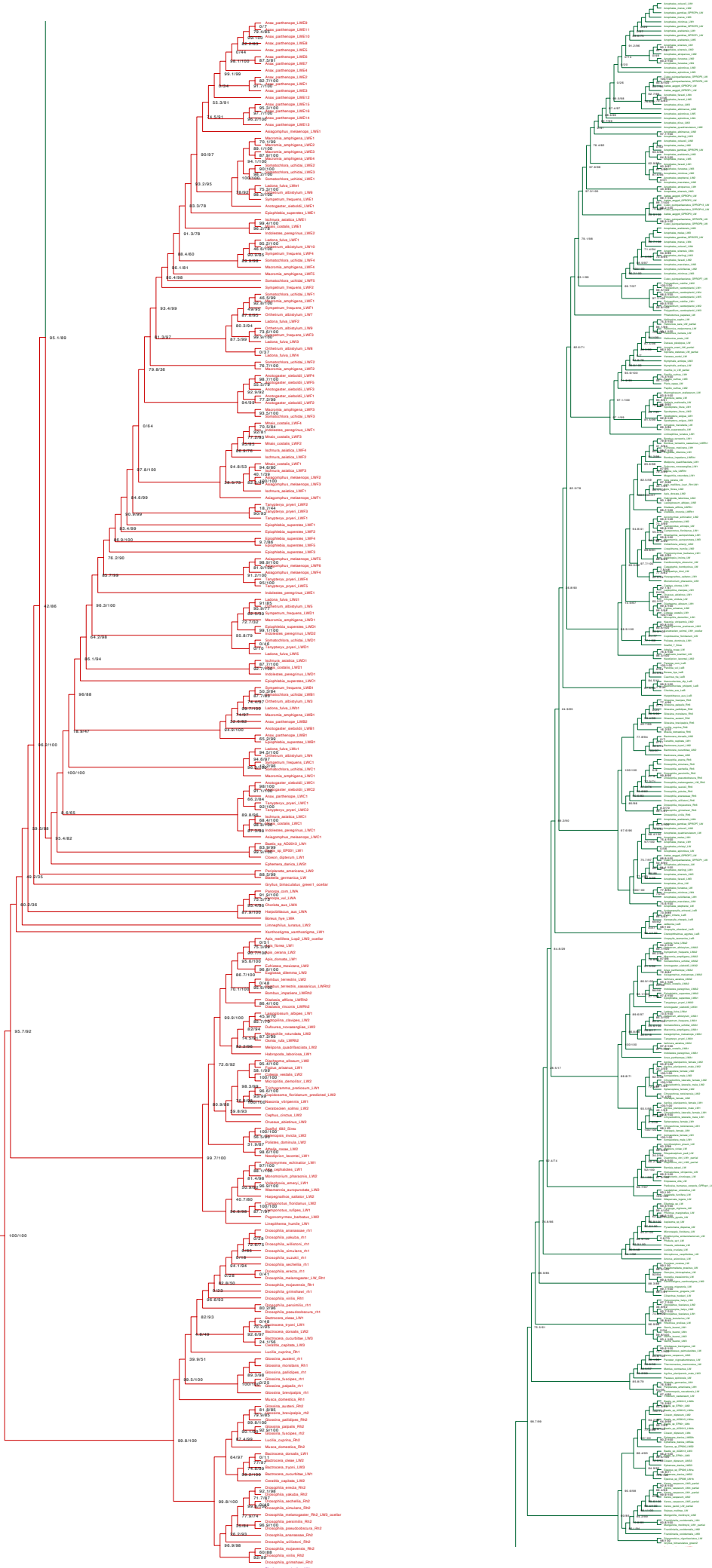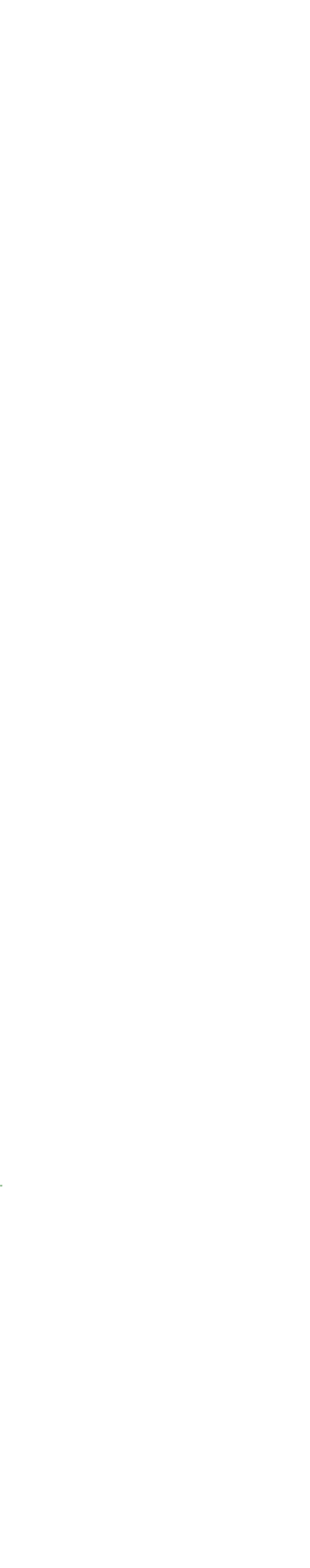

Supplement: Supplementary file 2 — Additional file 2: Fig. S1. Complete maximum-likelihood tree of 1000 insect visual opsin sequences. The LW2b, LW2a, SW and UV opsins are red, green, blue and purple, respectively. Node circles indicates UFbootstrap and SH-alrt value. [file 12862_2022_1960_MOESM2_ESM.pdf]
