# Supplementary material for: The evolution of insect visual opsin genes with specific consideration of the influence of ocelli and life history traits
Source: BMC Ecol Evol. 2022 Jan 7;22:2. doi: 10.1186/s12862-022-01960-8 (PMC8739693; doi:10.1186/s12862-022-01960-8)
Supplement: Supplementary file 3 — Additional file 3: File S1. Literature describing the number of ocelli in various insect orders. [file 12862_2022_1960_MOESM3_ESM.docx]

Literature used to determine the presence of three or less ocelli in insects.

**General**

Henze MJ, Oakley TH (2015) The dynamic evolutionary history of Pancrustacean eyes and opsins. Integrative and Comparative Biology 55:830–842. <https://doi.org/10.1093/icb/icv100>

Goodman LJ (1970) The structure and function of the insect dorsal ocellus. In: Beament JWL, Treherne JE, Wigglesworth VB (eds) Advances in Insect Physiology. Academic Press, pp 97–195

Mizunami M (1995) Functional diversity of neural organization in insect ocellar systems. Vision Research 35:443–452. <https://doi.org/10.1016/0042-6989(94)00192-O>

Taylor GK, Krapp HG (2007) Sensory systems and flight stability: what do insects measure and why? In: Casas J, Simpson SJ (eds) Advances in Insect Physiology. Academic Press, pp 231–316

Capinera JL (2008) Encyclopedia of Entomology. Springer Science & Business Media

Parry DA (1947) The function of the insect ocellus. Journal of Experimental Biology 24:211–219. <https://doi.org/10.1242/jeb.24.3-4.211>

Ribi W, Zeil J (2018) Diversity and common themes in the organization of ocelli in Hymenoptera, Odonata and Diptera. J Comp Physiol A 204:505–517. <https://doi.org/10.1007/s00359-018-1258-0>

Tauber CA, Tauber MJ, Albuquerque GS (2009). Encyclopedia of Insects. Elsevier, pp 695–707

**Hemiptera**

Armisén D, Rajakumar R, Friedrich M, et al (2018) The genome of the water strider Gerris buenoi reveals expansions of gene repertoires associated with adaptations to life on the water. BMC Genomics 19:832. <https://doi.org/10.1186/s12864-018-5163-2>

Burdfield-Steel ER, Shuker DM (2014) The evolutionary ecology of the Lygaeidae. Ecol Evol n/a-n/a. <https://doi.org/10.1002/ece3.1093>

Hamilton KGA (1981) Morphology and evolution of the Rhynchotan head (Insecta: Hemiptera, Homoptera). Can Entomol 113:953–974. <https://doi.org/10.4039/Ent113953-11>

Mcneill CA, Allan SA, Koehler PG, et al (2016) Vision in the common bed bug *Cimex lectularius* L. (Hemiptera: Cimicidae): eye morphology and spectral sensitivity: Bed bug vision. Med Vet Entomol 30:426–434. <https://doi.org/10.1111/mve.12195>

Shcherbakov D, Popov Y (2002) Order Hemiptera Linné, 1758. The bugs, cicadas, plantlice, scale insects, etc. pp 143–157

**Thysanoptera**

Gunawardana DN, Li D, Masumoto M, et al (2017) Resolving the confused identity of *Frankliniella panamensis* (Thysanoptera: Thripidae). Zootaxa 4323:125–131. <https://doi.org/10.11646/zootaxa.4323.1.10>

**Psocodea**

Capinera JL (2008) Encyclopedia of Entomology. Springer Science & Business Media

**Lepidoptera**

Dickens JC, Eaton JL (1973) External Ocelli in Lepidoptera Previously Considered to be Anocellate. Nature 242:205–206. <https://doi.org/10.1038/242205a0>

Dow MatthewA, Eaton JohnL (1976) Fine structure of the ocellus of the cabbage looper moth (Trichoplusia ni). Cell Tissue Res 171:. <https://doi.org/10.1007/BF00220243>

**Trichoptera**

Holzenthal RW, Blahnik RJ, Prather AL, Kjer KM (2007) Order Trichoptera Kirby, 1813 (Insecta), Caddisflies. Zootaxa 1668:639–698. <https://doi.org/10.11646/zootaxa.1668.1.29>

**Diptera**

Pollock JA, Benzer S (1988) Transcript localization of four opsin genes in the three visual organs of Drosophila; RH2 is ocellus specific. Nature 333:779–782. <https://doi.org/10.1038/333779a0>

Sabat D, Priyadarsini S, Mishra M (2016) Understanding the structural and developmental Aapect of simple eye of Drosophila: the ocelli. Journal of Cell Signaling 1:1–10. <https://doi.org/10.4172/2576-1471.1000109>

**Mecoptera**

Böhm A, Meusemann K, Misof B, Pass G (2018) Hypothesis on monochromatic vision in scorpionflies questioned by new transcriptomic data. Sci Rep 8:9872. <https://doi.org/10.1038/s41598-018-28098-2>

Manwaring KF, Whiting MF, Wilcox E, Bybee SM (2016) A study of common scorpionfly (Mecoptera: Panorpidae) visual systems reveals the expression of a single opsin. Organisms Diversity & Evolution 16:201–209. <https://doi.org/10.1007/s13127-015-0241-7>

**Siphonaptera**

Taylor SD, de la Cruz KD, Porter ML, Whiting MF (2005) Characterization of the Long-Wavelength Opsin from Mecoptera and Siphonaptera: Does a Flea See? Molecular Biology and Evolution 22:1165–1174. <https://doi.org/10.1093/molbev/msi110>

**Coleoptera**

Crowson RA (2013) The Biology of the Coleoptera. Academic Press

Tierney SM, Cooper SJB, Saint KM, et al Opsin transcripts of predatory diving beetles: a comparison of surface and subterranean photic niches. Royal Society Open Science 2:140386. <https://doi.org/10.1098/rsos.140386>

Leschen R a. B, Beutel RG (2004) Ocellar atavism in Coleoptera: plesiomorphy or apomorphy? Journal of Zoological Systematics and Evolutionary Research 42:63–69. <https://doi.org/10.1046/j.0947-5745.2003.00241.x>

**Strepsiptera**

Pohl H, Niehuis O, Gloyna K, et al (2012) A new species of Mengenilla (Insecta, Strepsiptera) from Tunisia. Zookeys 79–101. <https://doi.org/10.3897/zookeys.198.2334>

Cook JL (2014) Review of the Biology of Parasitic Insects in the Order Strepsiptera. Comparative Parasitology 81:134–151. <https://doi.org/10.1654/4723.1>

James M, Nandamuri SP, Stahl A, Buschbeck EK (2016) The unusual eyes of Xenos peckii (Strepsiptera: Xenidae) have green- and UV--sensitive photoreceptors. J Exp Biol 219:3866–3874. <https://doi.org/10.1242/jeb.148361>

Buschbeck EK (2005) The compound lens eye of Strepsiptera: morphological development of larvae and pupae. Arthropod Structure & Development 34:315–326. <https://doi.org/10.1016/j.asd.2005.04.002>

**Neuroptera**

Tauber CA, Tauber MJ, Albuquerque GS (2009) Neuroptera. In: Encyclopedia of Insects. Elsevier, pp 695–707

**Raphidioptera**

Eidissen SE (2015) Inocellia crassicornis (Schummel, 1832) (Raphidioptera) new to Norway. 4

**Hymenoptera**

Ross HH (1937) A generic classification of the Nearctic sawflies (Hymenoptera, Symphyta). [s.n.], Urbana, Ill.,

Weiblen GD (2002) How to be a Fig Wasp. Annu Rev Entomol 47:299–330. <https://doi.org/10.1146/annurev.ento.47.091201.145213>

Wang B, Xiao J-H, Bian S-N, et al (2013) Evolution and Expression Plasticity of Opsin Genes in a Fig Pollinator, Ceratosolen solmsi. PLOS ONE 8:e53907. <https://doi.org/10.1371/journal.pone.0053907>

Kazmi* SI, Hayat M (1998) Revision of the Indian Copidosomatini (Hymenoptera: Chalcidoidea: Encyrtidae). Oriental Insects 32:287–362. <https://doi.org/10.1080/00305316.1998.10433781>

Lue C-H, Driskell AC, Leips J, Buffington ML (2016) Review of the genus Leptopilina (Hymenoptera, Cynipoidea, Figitidae, Eucoilinae) from the Eastern United States, including three newly described species. JHR 53:35–76. <https://doi.org/10.3897/jhr.53.10369>

Guignard Q, Spaethe J, Slippers B, et al (2021) Evidence for UV-green dichromacy in the basal hymenopteran Sirex noctilio (Siricidae). Sci Rep 11:15601. <https://doi.org/10.1038/s41598-021-95107-2>

Yilmaz A, Lindenberg A, Albert S, et al (2016) Age-related and light-induced plasticity in opsin gene expression and in primary and secondary visual centers of the nectar-feeding ant *Camponotus rufipes*. Developmental Neurobiology 76:1041–1057. <https://doi.org/10.1002/dneu.22374>

Kelber A, Jonsson F, Wallén R, et al (2011) Hornets can fly at night without obvious adaptations of eyes and ocelli. PLOS ONE 6:e21892. <https://doi.org/10.1371/journal.pone.0021892>

Kerfoot WB (1967) Correlation between ocellar size and the foraging activities of bees (Hymenoptera; Apoidea). The American Naturalist 101:65–70. <https://doi.org/10.1086/282470>

**Blattodea**

Krishna K (2012) Biology of Termites. Elsevier

**Phasmatodea**

Buckley TR, Myers SS, Bradler S (2014) Revision of the stick insect genus Clitarchus Stål (Phasmatodea: Phasmatidae): new synonymies and two new species from northern New Zealand. Zootaxa 3900:451. <https://doi.org/10.11646/zootaxa.3900.4.1>

**Orthoptera**

Henze MJ, Dannenhauer K, Kohler M, et al (2012) Opsin evolution and expression in arthropod compound eyes and ocelli: insights from the cricket *Gryllus bimaculatus*. BMC Evolutionary Biology 12:163. <https://doi.org/10.1186/1471-2148-12-163>

Rence BG, Lisy MT, Garves BR, Quinlan BJ (1988) The role of ocelli in circadian singing rhythms of crickets. Physiological Entomology 13:201–212. <https://doi.org/10.1111/j.1365-3032.1988.tb00924.x>

Wilson M (1978) The functional organisation of locust ocelli. J Comp Physiol 124:297–316. <https://doi.org/10.1007/BF00661380>

**Ephemeroptera**

Almudi I, Vizueta J, Wyatt CDR, et al (2020) Genomic adaptations to aquatic and aerial life in mayflies and the origin of insect wings. Nature Communications 11:2631. <https://doi.org/10.1038/s41467-020-16284-8>

**Odonata**

Futahashi R, Kawahara-Miki R, Kinoshita M, et al (2015) Extraordinary diversity of visual opsin genes in dragonflies. Proceedings of the National Academy of Sciences 112:E1247–E1256. <https://doi.org/10.1073/pnas.1424670112>

Chappell RL, DeVoe RD (1975) Action spectra and chromatic mechanisms of cells in the median ocelli of dragonflies. J Gen Physiol 65:399–419. <https://doi.org/10.1085/jgp.65.4.399>
